# Socio-economic status is a social construct with heritable components and genetic consequences

**DOI:** 10.1038/s41562-025-02150-4

**Published:** 2025-03-26

**Authors:** Abdel Abdellaoui, Hilary C. Martin, Martin Kolk, Adam Rutherford, Michael Muthukrishna, Felix C. Tropf, Melinda C. Mills, Brendan Zietsch, Karin J.H. Verweij, Peter M. Visscher

**Affiliations:** 1 Department of Psychiatry, Amsterdam UMC, University of Amsterdam, Amsterdam, the Netherlands; 2 Human Genetics Programme, Wellcome Sanger Institute, Wellcome Genome Campus, Hinxton, UK; 3 Demography Unit, Department of Sociology, Stockholm University, Stockholm, Sweden; 4 Institute for Futures Studies, Stockholm, Sweden; 5 Department of Genetics, Evolution and Environment, University College London, London, UK; 6 Department of Psychological and Behavioural Science, London School of Economics and Political Science, London, UK; 7 Data Science Institute, London School of Economics, United Kingdom; 8 STICERD, London School of Economics, United Kingdom; 9 Centre for Longitudinal Studies, University College London, London, UK; 10 Department of Sociology, Purdue University, West Lafayette, USA; 11 Leverhulme Centre for Demographic Science, Nuffield Department of Population Health and Nuffield College, University of Oxford, Oxford, UK; 12 Department of Economics, Econometrics and Finance, Faculty of Economics and Business, University of Groningen, Groningen, the Netherlands; 13 Department of Genetics, University Medical Centre Groningen, Groningen, the Netherlands; 14 Centre for Psychology and Evolution, School of Psychology, University of Queensland, Brisbane, Australia; 15 Big Data Institute, Li Ka Shing Centre for Health Information and Discovery, Nuffield Department of Population Health, University of Oxford, Oxford, UK; 16 Institute for Molecular Bioscience, University of Queensland, Brisbane, Australia

## Abstract

In civilizations, individuals are born into or sorted into different levels of socio-economic status (SES). SES clusters in families and geographically, and is robustly associated with genetic effects. Here, we first review the history of scientific research on the relationship between SES and heredity. We then discuss recent findings in genomics research in light of the hypothesis that SES is a dynamic social construct that involves genetically influenced traits that help in achieving or retaining a socio-economic position, and can affect the distribution of genes associated with such traits. Social stratification results in people with differing traits being sorted into strata with different environmental exposures, which can result in evolutionary selection pressures through differences in mortality, reproduction, and non-random mating. Genomics research is revealing previously concealed genetic consequences of the way society is organized, yielding insights that should be approached with caution in pursuit of a fair and functional society.

Human societies throughout history have often been stratified by socio-economic status (SES), with different groups of people having access to different levels of power, prestige, wealth, health, freedom, and overall quality of life (see Box 1).^4^ While some believe that, in a meritocratic system, inequality can serve as an incentive for individuals to be more productive, inequality – or an excess thereof – is also considered disruptive, with detrimental effects on social cohesion.^5–7^ Rising inequality is generally accompanied by growing disparities in mental and physical health.^7,8^ While in recent times, inequality between countries has broadly declined, inequality within many countries has been increasing, especially since the 1980s.^9,10^ Social inequality is an inherently societal phenomenon driven by cultural, structural, economic, political, and technological forces, although, as we show here, it is also associated with genetic variation. While behavioral genetics research is actively examining the relationship between genetics and SES,^11^ most studies within the broader social sciences aiming to understand social inequalities tend to focus on societal factors.^9,12^ By not including genetic effects, a significantly contributing force is omitted that may be increasing in importance due to recent societal changes. When acknowledging these genetic effects, however, it is important to tread with caution. Recent history has shown that attempts to control the genetic make-up of populations – in the form of eugenics – can result in serious violations of human rights, including limiting access to education and labor markets, involuntary sterilization, infanticide, and genocide.^13–15^

In the first half of this article, we review the history of social stratification and the scientific study of its relationship with DNA. We summarize recent developments in genomics research that have provided us with a wealth of data on the relationships between genetic effects and socio-economic outcomes, albeit overwhelmingly in populations of European ancestry and with a bias towards individuals with higher SES (Box 2). In the second half of this article, we discuss how these new data could be interpreted in the context of SES as a dynamic social construct that could exert natural selection pressures on genes associated with socially advantageous traits.

## History of Social Stratification and the Science of Heredity

### Social Stratification

Civilizations are generally defined as complex societies with urban development, some form of government, symbolic systems of communication (e.g., writing), and social stratification.^16,17^ While there are no known civilizations without some form of social stratification, it is not a defining characteristic of human societies in general. Most of the hunter-gatherer societies known today are relatively egalitarian.^4,18,19^ Social stratification became more pronounced with the rise of larger and more complex human societies that arose during developments of the Neolithic era starting about 12,000 years ago.^20^ Broadly speaking, the gradual shift from hunter-gatherer to sedentary agricultural societies enabled surplus resource accumulation, which led to an increase in population size and division of labor.^21^ This allowed for different levels of prestige to develop through job specialization and more unequal accumulation of possessions. In numerous societies, elite classes arose that gained control over food supplies, land, means of production, and the labor of much of their population. In many instances, legal and structural systems developed over time that codified these social hierarchies, reinforcing the power and privilege of the elites and cementing the stratification within societies.^22^

Human history has known relatively rigid social stratification systems with little movement between socio-economic levels, often maintained through religious beliefs that legitimized the divine mandate of rulers.^23^ Social status was often ascribed, with children inheriting their parents’ status. Many of the phenomena discussed in this article apply to systems that show at least some degree of merit-based social mobility. The earliest recorded example of a formal merit-based system emerged in 6^th^ century BC China, where Confucian scholars advocated education for all, and introduced the notion that those who govern should do so based on individual merit rather than inherited status.^24^ Such meritocratic principles were later applied by Genghis Khan in the 13th century, selecting leaders based on ability rather than family.^25^ European scholars translated Confucian’ texts in the 17^th^ century, exposing them to alternative perspectives on governance and social organization. These Confucian ideals probably contributed to the intellectual milieu of the Enlightenment movement, where merit-based social systems gained prominence.^26^ The medieval European estate system, where noble or common status was largely determined by birth, made way for socio-economic orders that aimed for more equal opportunities. As the Industrial Revolution unfolded, bringing increased production, economic growth, and social change, a modern, more merit-based socio-economic system began to emerge, transitioning to a new social order that could accommodate an ever-expanding population, while also increasing a visible underclass.

Compared to many pre-industrial socio-economic orders, merit-based hierarchies increase opportunities across the population, allocate talent more efficiently, and stimulate progress through competition between people and between firms. The term ‘meritocracy’, however, was originally coined in a negative light in the 1958 satire “The Rise of the Meritocracy” by Michael Young.^27^ This book describes a dystopian future, in which meritocracy has led to a newly stratified society, replacing an aristocracy of birth by an aristocracy of talent, with a disenfranchised lower class of the less meritorious. If behaviors associated with merit (e.g., intelligence, persistence, creative talent) are partly heritable, variation in genetics within families could still facilitate social mobility. Enduring accumulation of resources within families however could limit this mobility, gradually reverting meritocracy back towards an aristocracy of birth.

### Heredity

Contemporary research shows SES to be central to social stratification, focusing on intergenerational transmission of education, occupation, class, earnings, and wealth, and variations across countries, history, gender, and ethnicity.^28–31^ Social science research on social stratification and intergenerational transmission of SES has largely ignored or actively resisted the study of its relationship with genetic factors, partly due to ethical concerns and historical misuse of genetics in social policy.^32^ To better understand this oversight, it is important to consider the history of genetics research and its societal impact.

Scientific research exploring connections between genetics and socio-economic success has a turbulent and controversial history. During the 16^th^ century, early ideas about biological heredity were influenced by legal concepts of cross-generational inheritance of property and wealth.^33^ The concept of heritability began to be formalized in the 19th and early 20th centuries in the light of the work of Mendel and Darwin, whose work revealed the laws of inheritance and mechanisms of evolution. Charles Darwin’s half-cousin, Francis Galton, explored the heritability of traits linked to merit and socio-economic success in his book, *Hereditary Genius* (1869). In this period, a prelude to the emergence of the field of genetics, Galton and his followers put more emphasis on ‘nature’ than nurture. In his book, Galton applied statistics to show that offspring of “eminent” figures had a higher chance of succeeding in what were perceived to be high-profile professions.^34^ Inspired by these findings, Galton became a proponent of improving what became known as the ‘genetic quality’ of a population through selective parenthood, thus initiating and spearheading the emerging eugenics movement.^35^ This movement became widely supported in many countries across the world and across the political spectrum by established intellectuals and medical authorities.^13^ Eugenics proponents intended to explore and enact policies that would increase the overall wellbeing of majority populations or dominant social groups, but inevitably at the expense of others who were deemed economically costly or socially undesirable, and suffered stigmatization and persecution as a result.^15^ In many cases, eugenic ideas resulted in state-sponsored violence against marginalized groups, primarily via enforced or coerced sterilization.^36–38^ The destructive power of the eugenics movement reached genocidal levels in the Second World War, after which its public support declined. The legacy of involuntary sterilization is still detectable, with population register data revealing that individuals categorized with severe mental and physical disabilities (up to 1970 in Finland and 1976 in Sweden) often remained childless.^39^ Enforced or coercive sterilization continues in several countries to this day, including China and India, the two most populous countries on earth, often targeting lower socio-economic groups as a means of population control.^15^ In the second half of the 20^th^ century, the scientific field of heredity did become largely decoupled from social applications in most countries and made progress through decades of twin and family studies.^40^

When it comes to socio-economic success, merit in contemporary industrialized societies typically involves strong performance in the educational system and/or labor market, both of which have intelligence – defined and measured in various ways – as one of their strongest predictors, alongside non-cognitive predictors such as parent’s socioeconomic status and individual-level traits such as conscientiousness.^41–45^ Intelligence was the first trait studied using the classical twin method,^46,47^ which estimates heritability by comparing the resemblance of identical and fraternal twins. The considerable heritability of intelligence, i.e., the extent to which genetic differences explain individual differences within a population, and its increase from childhood (∼.43) to adulthood (∼.65), have become among the most replicated findings in twin research.^40,48,49^ A change in heritability can occur because genetic influences change over time or, more likely, because the variance in or influence of environments change.

**Figure 1:**
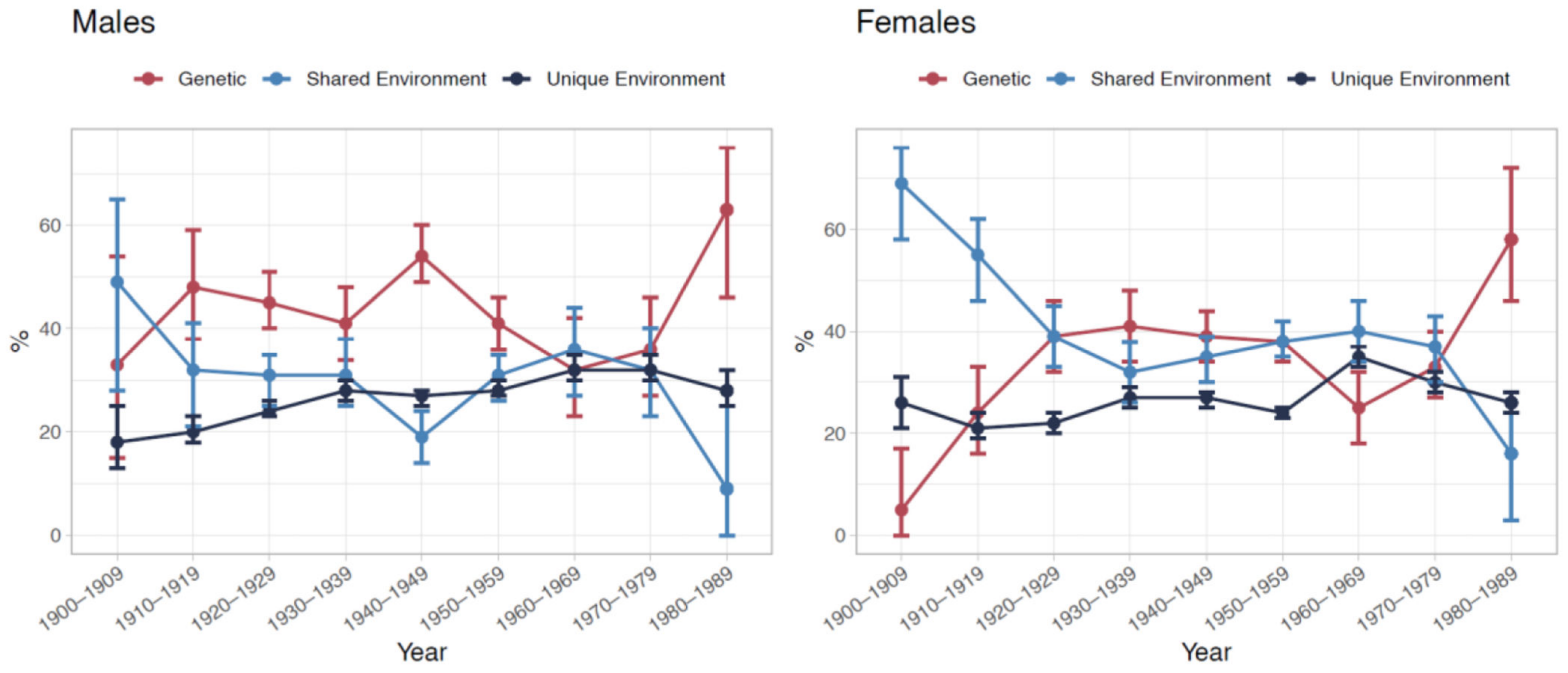
Changes in the heritability of educational attainment over time in Europe. The Figures show the percentage of variation in educational attainment explained by genetic and environmental influences with 95% confidence intervals, as estimated in a meta-analysis of 28 European twin cohorts. Shared environmental influences refer to environmental factors that make siblings more similar to each other, while unique environmental influences are factors that do not, and also include measurement error. In twin studies, shared environmental influences can be overestimated at the expense of genetic influences when assortative mating occurs, as it increases the genetic similarity between siblings, mimicking shared environmental influences. The Figure is based on data from Table 2 from Silventoinen et al (2020).^51^

Twin and family studies suggest that the heritability of traits affecting socio-economic outcomes varies with societal equality. Theoretically, equalizing opportunities would reduce heritability estimates if the genetic correlates of SES operate through traits associated with inherited privilege or social biases, rather than through traits that enhance an individual’s ability to perform in a meritocratic system. If genetics mainly correlate with structural factors, such as ancestry-linked access to resources, power, or social networks, then reducing these inequalities should weaken the correlation between genetics and SES. Alternatively, if genetic correlates of SES tend to reflect traits that improve an individual’s competence in domains that influence social or economic mobility – like cognitive or problem-solving abilities – then reducing environmental barriers should increase heritability by making genetic differences more predictive of life outcomes. In welfare states with more equal opportunities and high intergenerational mobility, the heritability of education has seemed to increase, primarily due to reduced environmental influences and a relative constancy of genetic factors, increasing the relative contribution of genetics to the total variance.^50^ In Europe, the heritability of educational attainment for women grew from 5% for those born 1900-1909 to 58% by 1980-1989 (Figure 1),^51^ possibly due to improving educational systems and reduced societal barriers.

### The Genomics Era

Advances in genotyping technologies in the 21st century enabled genome-wide association studies (GWASs), first applied in 2005 after being proposed in 1996.^52–55^ In a GWAS, millions of genetic variants capturing most common genetic variation in a population are measured, and the effect of each variant on a trait of interest is estimated. The effects of individual genetic variants on complex traits turned out to be hard to distinguish from noise, due to their extremely small effect sizes and the heavy multiple testing burden. Pooling data from many cohorts has enabled GWAS consortia to reach sample sizes of millions, identifying tens of thousands of variants linked to hundreds of physical, mental, and behavioral traits.^56^

The first large-scale GWAS on educational attainment was published in 2013,^57^ conducted in ∼125 thousand individuals, identifying only three associated genetic variants explaining ∼0.02% of individual differences – a small harvest for such a large study, but enough to prompt further increases in sample size. Subsequent larger-scale GWASs reached up to 3 million individuals, identifying thousands of significantly associated variants.^1,58,59^ GWASs on income and occupational status, each with sample sizes in the hundreds of thousands, found hundreds of associations, with very similar polygenic signals across these SES indicators.^60–62^ Recent evidence indicates that a substantial portion of the signals detected in GWASs on SES-related traits may be confounded by ancestry differences (i.e., population stratification), which could affect downstream analyses (Box 2).^63^ Genetic correlations (*r*_g_) estimated via methods like LD Score Regression (which leverages correlational patterns of genetic variants to adjust for population stratification)^64^ are more likely to reflect the portion of the GWAS signal that is less affected by these confounds.^2^ This less-confounded part of the income GWAS is consistent across Western countries, including the UK, Netherlands, Norway, Estonia, and the US (average *r*_g_ = 0.88).^62^ Recently, the first educational attainment GWAS was conducted in a large East Asian dataset – South Koreans and Taiwanese – which, after adjusting for population stratification, found a remarkably similar genetic signal to the European GWASs (*r*_g_ = 0.87).^65^

As with many behavioral traits, increasing sample sizes reveal progressively smaller effect sizes, in line with the infinitesimal model – a concept that builds upon the early foundations in quantitative genetics conceptualized by R.A. Fisher a century earlier.^66^ Modern GWASs confirm that complex traits are influenced by a large number of genetic variants, each with a small effect,^67,68^ but collectively explaining substantial variance. One way to harness the power of the aggregate genetic effects is through so-called polygenic scores, in which the alleles an individual carries are weighted by their estimated effects on a trait and then summed to produce genetic predictors. Many of the studies discussed below are based on polygenic scores constructed from the educational attainment GWASs, as these currently provide the most predictive scores due to their statistical power. Polygenic scores for educational attainment from a 3-million-individual GWAS explain 12-16% of educational differences in Europeans,^59^ with about half attributable to the clustering of economic resources in families and assortative mating (Box 3).^63^ Larger GWASs are expected to improve precision of genetic effect estimates and thereby the predictive power of polygenic scores, although this depends on similarities between the discovery GWAS and the target datasets.^69^ These polygenic scores capture part of the heritability of socio-economic outcomes, reflecting biological, social, and demographic processes, and correlated environmental exposures (Box 3). Genetic scores computed from birth with predictive power on future socio-economic outcomes hold potential value for research as well as policy development. Currently, however, their predictive power arises from a largely elusive combination of underlying traits and environmental influences.^70^ Analyses of GWAS signals for SES outcomes using enrichment tools applied to diseases show the strongest enrichment in brain and neuronal processes,^1,62^ consistent with the role for cognitive and behavioral traits in SES. Rather than solely striving to expose underlying biology and perfect genetic effect size estimates, the field has great potential when striving to understand what causes genetic effects to vary across different environments and populations—a direction we explore in the following sections on socio-economic status as a selection pressure.

## Social Stratification as a Natural Selection Pressure

When Charles Darwin presented his theory of evolution by natural selection,^71^ he wrote that nature selects for adaptations that give organisms an advantage in the three struggles of life: with the physical environment, with other species, and with members of one’s own species. Over time, humans probably reduced the first two selection pressures while intensifying the third through social and economic competition, where winners are rewarded with the favorable social and environmental circumstances that come with higher SES.

GWASs on socio-economic outcomes produce polygenic signals that contain genetic effects on a mixture of traits and environmental effects.^70^ Here, we describe social forces that bind these traits and environmental effects together. In human societies, people become sorted into more favorable or less favorable environments, depending on a combination of their inherited privileges and their performance in the socio-economic system. This sorting could create selection pressures through differential mortality, reproductive success, and mate choice. SES is a dynamic social construct which could target different associated traits and genes across time and space. In more merit-based systems, positive selection would probably act on genes associated with attributes and skills deemed beneficial by that particular society, assuming these translate to greater reproductive success; however, as we explain further below in the section *Reproductive Success*, in practice, the direction of the selection effect may have varied over time, and there can be non-linear effects.^72^ In particular, formal education and the introduction of money as a standardized medium of exchange could be expected to have sharpened this selection pressure. An educational system is a relatively efficient way to nurture talents and stratify the population based on those talents at a relatively early age, while money offers a more efficient way to keep score of a person’s “earned” SES compared to earlier barter systems or reliance on resources like land.

Historically, varying environmental and social conditions impacted mortality and reproductive success, leading to complex patterns in the genetic architecture of complex traits. The highly polygenic and pleiotropic nature of complex traits, in addition to complex population structures, complicate detection of past selection pressures at the DNA level.^73^ Selection pressures on complex traits are dispersed across many minor effects. The most pervasive selection pressures detectable in genetic data across most complex traits, including educational attainment, are negative selection pressures, indicated by genetic variants with larger effects being kept at lower frequencies.^74,75^ This pattern reflects a consistent constraint on extreme phenotypes (stabilizing selection), flattening the distribution of effect sizes and resulting in high polygenicity.^76^ Thousands of genes show a strong signature of historical negative selection against damaging variants,^77–79^ and in contemporary populations, rare damaging variants in these genes have been associated with lower intelligence and educational attainment.^80–82^ Modern selection pressures are expected to be detectable through associations between polygenic effects and patterns of mortality, reproduction, and non-random mating, all of which appear to be strongly driven by SES-related outcomes that vary across time and space, as discussed in more detail below.

### Genes, Geography, and Mortality

After the advent of agriculture, societies became larger and more complex.^83^ Over the millennia of the Neolithic era, population growth led to urbanized settlements that covered geographic regions orders of magnitude larger than their predecessors. Different populations covered different geographical areas, and within populations and cities, socio-economic strata covered different regions.^84^ The largest patterns of genetic variation align with differences between ancestral populations, which correlate strongly with geography, because individuals tend to reproduce with people who live closer to them geographically (see *Population Stratification* in Box 2).^85–87^ If socio-economic outcomes are based on heritable merit-based outcomes, such as the performance in an educational system, it would be expected that the genes associated with these outcomes would show regional differences within populations as well.

Analyses of the geographic distribution of polygenic scores for a variety of behavioral, cognitive, and health-related outcomes across Great Britain and across Estonia revealed that, once ancestry differences were minimized, the strongest regional differences were in educational attainment polygenic scores.^3,88^ These geographic differences aligned with regional socio-economic differences; in Great Britain, lower polygenic scores clustered in economically disadvantaged coal mining areas,^3^ and in Estonia, higher polygenic scores were more concentrated in the two prospering university towns.^88^ Migration contributed to these differences by increasing the geographic clustering of polygenic scores throughout the 20th century while simultaneously reducing the correlation between ancestry and geography, as individuals with higher polygenic scores tended to move to more prosperous regions.^3,88^

**Figure 2:**
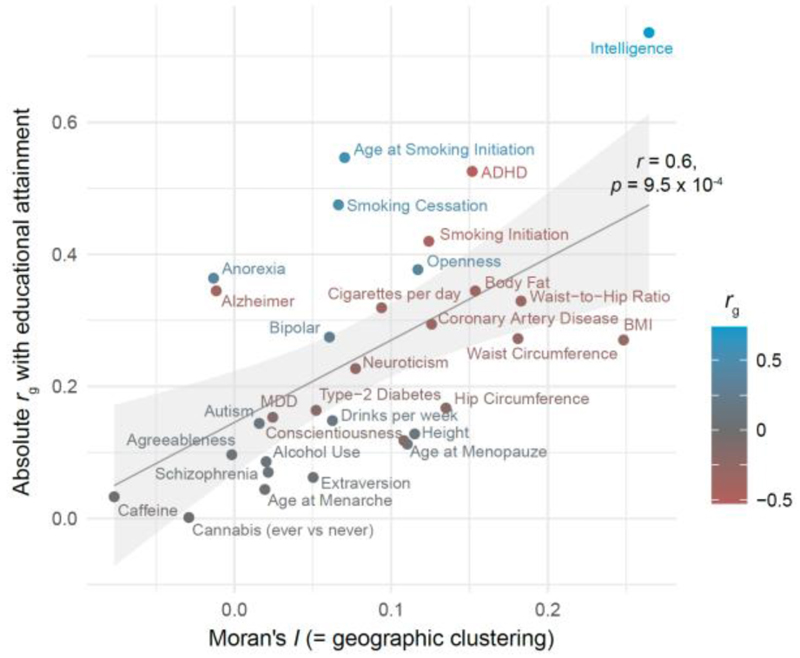
Traits that show higher genetic correlations with educational attainment tend to show stronger regional differences. Genetic correlations (r_g_) can vary between -1 (100% shared variance due to the same genetic effects in the opposite direction) through 0 (no overlap in genetic effects) to 1 (100% of shared variance due to the same genetic effects in the same direction). Y-axis indicates the absolute genetic correlation of the trait with educational attainment (EA) (Lee et al, 2018)^1^, excluding all British. Genetic correlations were computed with LD Score Regression.^2^ X-axis shows the Moran’s I, a measure of geographic clustering, of 31 polygenic scores in ∼320k individuals in Great Britain. The Moran’s I of the educational attainment polygenic score is 0.6 (not shown). This Figure was adapted from Supplementary Figure 5 in Abdellaoui et al (2019)^3^.

Polygenic scores for educational attainment and other SES outcomes are derived from GWASs estimating genetic effects on traits relevant to contemporary socio-economic systems. We can get a sense of the contribution of the underlying traits by estimating genetic correlations (*r*_g_) between traits. The genetic correlation between educational attainment, income, and occupational status are ∼0.9 in many developed countries, suggesting that mostly the same genetic signal is being picked up for all three traits, namely that of socio-economic status in those specific societies.^60–62,65^ Of all traits investigated so far, intelligence shows the highest genetic correlation with socio-economic status (*r*_g_ with educational attainment, income, and occupational status ∼0.7); personality dimensions and mental and physical health outcomes also share a significant portion of their genetic effects with these SES outcomes.^1,60–62^ Polygenic scores of traits that contribute more to socio-economic success tend to show stronger regional differences in Great Britain (Figure 2) and in Estonia than traits that are not.^3,88^

**Figure 3:**
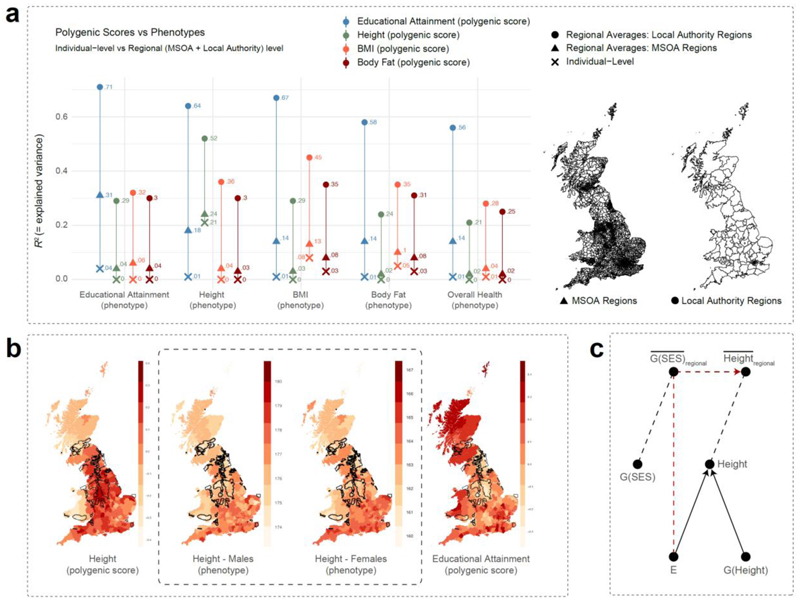
Polygenic prediction of average phenotypes per region likely captures environmental influences. Polygenic scores for educational attainment (EA) capture environmental effects on a regional level that are not visible when examining individual-level data. Panel **(a)** shows this based on ∼320k unrelated UK Biobank participants of European descent. The polygenic score for height explains 21% of individual differences in height, while the polygenic score for EA explains only 1% of individual differences in height. When we consider the average scores per region, however, the polygenic score for EA explains more regional differences in height (64%) than the polygenic score for height does (52%), presumably because the regional average of the EA polygenic score better captures regional differences in poor versus rich environments, and these affect height. Panel **(b)** displays the geographic distributions of regional averages of polygenic scores for height and EA, alongside the regional average for phenotypic height. Black lines denote coal mining regions, where environmental circumstances associated with socio-economic deprivation tend to cluster with polygenic scores for EA. Without such clustering, coal mining regions would be among the taller regions of the country, which phenotypically they are not. For statistical analyses regarding these regional differences and the migration herein, see Abdellaoui et al. (2019)^85^. The hypothesized causal diagram in panel **(c)** illustrates environmental influences on height (E) that cluster regionally with genetic influences associated with SES (G(SES)), making the regional average of those genes ${\bar{\left(G(SES\right)}}_{regional\;})$ predictive of the regional average of phenotypic height $\left({\bar{\;Height\;}}_{regional\;}\right)$. G(Height) denotes the genetic influences on height on an individual level (Height). For details on the data and QC for panels (**a**) and (**b**), see Abdellaoui et al 2022^91^; for details on polygenic score computation and geographic regions, see Abdellaoui et al, 2019^3^.

Genetic effects associated with educational attainment, income, or occupational status are a patchwork of many underlying heritable outcomes, but these effects, as estimated in a GWAS, are also intertwined with environmental effects. Environmental factors are diverse and include cultural, social, economic, and geographical contexts, ranging from societal structures like housing quality, dietary options, healthcare, and education systems to natural conditions such as air pollution. The increased efficiency with which populations in modern societies are stratified according to these heritable outcomes into different layers of environmental exposures leads to correlations between genes and environment that result in both ‘double advantages’ and ‘double disadvantages’. As a result, conducting a standard population-based GWAS on an SES-related outcome is partially equivalent to doing a GWAS on being born into a better environment and/or the ability to move to a better environment. Molecular genetic evidence shows that SES-related genetic effects and environmental influences cluster on both a family-level^63,89,90^ and regional level^3,91^, resulting in systematic differences in these environmental exposures. These environmental factors that correlate with genetics can cause additional (regional) differences in mental and physical health outcomes, such as substance use and BMI,^91^ but also more heritable traits, such as height (heritability ∼80%; Figure 3).

**Figure 4:**
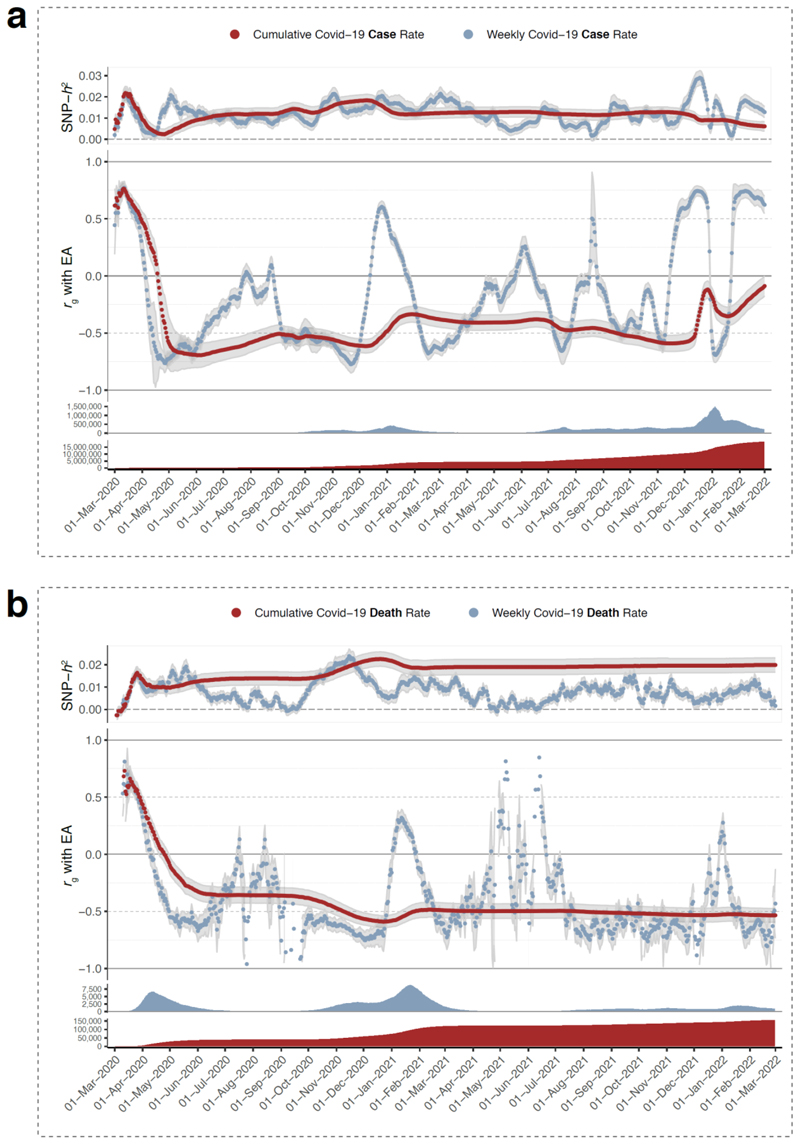
Genetic correlations show that Covid-19 infections and deaths in England originate in higher SES regions and spread more widely in lower SES regions. Panels **a** and **b** show data for Covid-19 cases and deaths respectively. The panels show results from a total of 2,924 regional GWASs (RGWASs; four per day - cases and deaths, cumulative and weekly - for 2 years, i.e., 731 days) performed on 1.2 million common single nucleotide polymorphisms (SNPs) from 396,042 individuals of European descent living in England. As opposed to a traditional GWAS, in an RGWAS the subjects are given the phenotype of the region they live in (315 regions), which often results in genetic signals associated with socio-economic outcomes due to their geographic clustering.^3^ Covid-19 data on the 315 regions were obtained from Public Health England. Each dot is one RGWAS (one day), for which either the weekly or the cumulative cases (**a**) or deaths (**b**) were analyzed as the phenotype. The upper panel shows the variation explained by all 1.2 million SNPs (the SNP-based heritability or SNP-h^2^). The large middle panel shows the genetic correlation (r_g_) of the genetic signal with the educational attainment (EA) GWAS from Lee et al (2018)^1^. The gray shaded areas around the points indicate 95% confidence intervals for both the SNP-h^2^ and genetic correlations. These genetic correlations shows several positive peaks, including at the start of the pandemic in March 2020 and around the start of the spread of the new and more contagious B1.1.7 variant in December 2020, both reflecting more infections in richer regions of the country (in or near London), after which the genetic correlation with education becomes negative again. The two bottom panels show the total number of cumulative and weekly Covid-19 cases (**a**) or deaths (**b**). For more information on quality control and statistical approaches used, see Abdellaoui (2020)^96^, where the same results were reported for cases only for the first ∼6 weeks of the Covid-19 pandemic.

People with genetic variants that make it easier for them to get a better education are more likely to move to better neighborhoods, while the people left behind are in worse living circumstances with higher mortality rates and greater risk for health problems like obesity, diabetes,^3^ and infectious diseases. Regional differences in Covid-19 infection and mortality rates, for instance, also show significant genetic correlations with SES (Figure 4), as it was easier for people in certain occupations, smaller households, and better housing to avoid the disease by working from home and to be physically better prepared for infection through healthy diet and exercise.^92^ Covid-19 was not likely to exert a strong selection pressure, because mortality was only high among those past the reproductive age. Previous pandemics, however, with higher mortality rates for younger people, such as the black death, smallpox, and the Spanish flu, had higher mortality in areas of lower SES as well.^93^ Besides the typical health consequences associated with lower-quality living conditions, deadly pandemics – whose frequency has increased since the advent of agriculture and cities^94,95^– could potentially impact allele frequencies of genetic variants that affect traits that are more consistently associated with social stratification.

### Reproductive Success

Natural selection affects allele frequencies through differences in reproductive success, influenced by differential mortality rates, fertility rates, and mate choice. Like other large mammals, human populations have historically faced density-dependent checks, where resource availability affected population density through mortality and fertility rates.^97^ In humans, these dynamics are likely intertwined with socio-economic status (SES).

A collection of ∼15,000 English men’s wills from the 16^th^ to the 20^th^ century showed a positive relationship between men’s income and net fertility in England, with the wealthiest leaving nearly twice as many offspring as the poorest.^98,99^ This was probably influenced by higher child mortality rate in lower SES groups,^99,100^ and greater mating opportunities for higher SES males, as women tend to prefer men with more resources across cultures with different mating systems, different levels of gender equality, and different religions.^101^ Pre-Industrial data from the 13^th^ to the 21^st^ century across multiple countries confirm this positive income-fertility relationship.^102^ As societies underwent significant transformations due to the Industrial Revolution, including changes in population density, urbanization, and industrialization, a general shift was observed across the world from a positive relationship to a negative relationship between income and reproductive success.^102^ Several explanations posed for this reversal include changes in child mortality, birth control, and women’s education and workforce participation.^102–104^ Studies on industrialized societies have shown sex differences in the association between wealth and fertility.^105^

In contemporary Western populations, including Great Britain, common genetic variants associated with higher SES show a negative correlation with offspring count.^72,106–109^ This implies a recent decline of these variants, despite their relationship with decreased mortality described in the previous paragraph. In contrast, rare genetic variants with stronger deleterious effects on intelligence, educational attainment, and income, negatively impact reproduction rates in Great Britain, especially in men.^82^ This may reflect a non-linear relationship between intelligence and reproductive success, with rare damaging variants being more predictive at the lower end of the intelligence spectrum.

More recently, the relationship between SES and reproductive success, which seems to be non-linear and sex-specific, seems to be reverting back to an overall positive one, both within and between a number of high-income countries; this is likely driven by multiple factors, such as an increased compatibility between women’s careers and families due to more favorable family policies and social norms, cooperative fathers, and more flexible labor markets.^110–112^

### Assortative Mating

Besides determining who propagates their genes, mate choice can also impact genetic variation when people choose partners similar to themselves. When people choose mates that resemble them for a trait – assortative mating – this affects genetic variation by widening the genetic distribution in the offspring population and increasing resemblance between family members.^66,113^ Humans tend to meet and choose partners similar to themselves in terms of ethnicity, religion, and socio-economic status.^114,115^ The strongest DNA similarity in spouses is for ancestry-related variation,^116^ followed by polygenic effects associated with educational attainment, which show the highest assortative mating levels among studied traits so far.^117–121^ Interestingly, the educational attainment associated loci show a higher correlation between spouses than expected based on the phenotypic spousal correlations. Possible explanations include additional assortative mating on the basis of more heritable underlying traits (e.g., intelligence), matching based on characteristics of both the mate and their family members, or inaccurate genetic effect estimates (see Boxes 2 and 3).^119,122,123^

While assortative mating on education may have been strengthened more recently by the increased heritability of educational attainment in women (Figure 1) and more women joining the workforce,^124^ studies into partnership markets around the Industrial revolution suggest that assortative mating on socio-economic outcomes is not a recent phenomenon.^125,126^ Data on 422,374 British inhabitants from 1600 to 2022 show substantial assortative mating and persistence of social status across generations despite significant social changes over time.^127^ In Norway, assortative mating continues to increase genetic similarities within families for genetic variants associated with educational attainment, and thus has not yet reached equilibrium.^120^ Equilibrium, where genetic similarities within families stabilize despite ongoing assortative mating, is expected to be achieved after many generations of stable assortative mating on this outcome. The ongoing increase in familial genetic similarity suggests a recent increase in assortative mating, potentially contributing to, or even due to, growing inequalities in contemporary Norwegian society.

Assortative mating probably increases with the geographic clustering of SES, since physical proximity increases the chance of finding a similar partner. SES has been clustering geographically since ancient times.^84^ Distances traveled with SES-related migration increased in recent times, which may have increased geographic clustering of SES-associated alleles,^3^ increasing assortative mating. If current rates of assortative mating on traits that influence SES persist or increase, this could further increase social inequalities on both an economic and genetic level over generations, making them harder to overcome. Assortative mating can also make economic factors become more intertwined with genetics over generations, as environmental advantages tied to SES influence mate selection. In both Great Britain and Norway, for example, it has been shown that earlier born siblings, who have a higher SES due to environmental factors, marry spouses with higher polygenic scores for educational attainment.^128^ The increasing correlation within individuals between SES-associated alleles across the genome, and between these alleles and environmental factors that influence SES, could have societal and evolutionary consequences, but also complicate the task of accurately quantifying genetic effects associated with SES (see Box 3).

## Conclusions

Social inequality has long been inherent in the way human societies are organized, arising because certain outcomes are more valued and rewarded than others, and reinforced by the familial clustering and transmission of status, resources, and genetic predispositions. Populations become stratified into social environments with differing levels of health risks, safety, and opportunity, leading to disparities in mental and physical health. When combining significant life quality differences between social layers with a certain amount of social mobility, it becomes desirable to climb the social ladder, stimulating many to try, but allowing only those with the most advantageous talents to succeed. Over time, this could influence the genetic make-up of populations through differential mortality, fertility, and non-random mating.

The strength and nature of these selection pressures vary across time and cultural contexts. Certain cognitive abilities may have conferred a more consistent advantage throughout our recent evolutionary history, particularly in more merit-based societies, which could have contributed to the increasingly complex societies that make humans such a unique primate. Technological advances may have impacted the effects of SES on genetic variation in multiple ways, including through improved and more accessible educational systems. These developments may, as a side-effect, have made humans more efficient at stratifying the population according to genetic talent, further inducing geographic clustering and assortative mating on SES-related genetic variants, potentially increasing genetic differences associated with social inequality. The more direct genetic effects, however, are significantly smaller than initially estimated in population-based genetic association studies, due to assortative mating and environmental influences that get entangled with genetic effects (see Box 3).

At the heart of these discussions is not a call for genetic intervention, but rather a call for a deeper understanding and awareness that our social structures are part of an evolving environment that, over time, shapes both social and genetic outcomes.^129,130^ The relationship between social stratification and genetic effects is complicated, consisting of a network of complex traits and environmental circumstances woven together by social and economic forces created by increasingly complex societies. While precise estimates of genetic effects are important, this field of research holds greater potential in uncovering how correlations between genes and social outcomes shift across societies and time.^50,51,131^ Processes like migration, mate choice, reproductive success, and mortality shape how populations develop and could create feedback loops that reinforce or reshape inequalities. Understanding these dynamics could help us trace societal structures of the past through traces left in the genome by, for example, assortative mating,^132^ reveal causes of present inequalities, and anticipate how inequalities might evolve with future social changes. This line of research can provide researchers across disciplines with a framework for studying the dynamic interplay between genetics, complex traits, and social organization. While mistakes from the past should keep us vigilant about the potential for harmful effects of genetically informed social policies, advances in interdisciplinary genomics research can help us better understand the processes underlying the way societies are organized and their consequences.

## Box 1 Definition of Socio-Economic Concepts.

- **Social Stratification:** The hierarchical organization of societies based on access to resources, power, and socio-economic status (SES), ranging from high to low. Group membership may or may not persist across generations.
- **Socio-Economic Status**: A social construct reflecting how society assigns value to certain outcomes, skills, traits, behaviors, achievements, and assets. SES captures and influences cultural norms within a specific society. In social science research, SES is typically described across four correlated dimensions:
  - *Income*: Total earnings from all sources (e.g., wages, bonuses, investments, rental income), typically averaged over multiple years and measured at the individual or household level.
  - *Education*: The formal acquisition of knowledge and skills through schooling, training, or self-directed learning. Education is both a cause and consequence of cognitive ability. It is often measured by years of attainment, earned credentials, and standardized test scores.
  - *Occupation*: Employment status, which can fluctuate over adulthood. Typically measured through occupational classes, continuous status scales, or ranking systems that combine education and occupational status (e.g., ISEI, SIOPS, CAMSIS)^60^.
  - *Wealth*: Net worth, defined as value of assets (e.g., property, financial holdings, possessions) minus liabilities. Wealth includes both self-generated and intergenerational components and is particularly difficult to measure at the top of the distribution.
- **(Intergenerational) Social Mobility:** The degree to which an individual’s SES rank correlates with that of their parents, reflecting changes in SES across generations.
- **Social Inequality:** The unequal distribution of resources, opportunities, and quality of life across individuals or households within a society at a given time.

## Box 2 Factors Affecting Estimation of Genetic Effects on Socio-Economic Outcomes (Part I).

### Sources of bias in estimating genetic effects

Genome-wide association studies (GWASs) can help illuminate genetic architectures of complex traits, but can also present a somewhat distorted view of this genetic architecture, particularly for behavioral and socio-economic outcomes. The three main sources of bias are:

- ***Population stratification***: The largest patterns of genetic variation within a population generally reflect ancestry differences, often from a more distant past. When cultural or environmental differences align with these genome-wide allele frequency differences, GWASs can produce false positive associations across the genome. While controlling for ancestry differences reduces bias, it is difficult to eliminate entirely.^63,133^ Uncontrolled population stratification could affect polygenic score differences between groups and effect sizes at individual loci, but should have less of an impact on genome-wide parameters estimated by LD Score Regression^64^ which explicitly deals with this problem. Controlling for ancestry could also obscure real genetic signals when the causal genetic variants themselves correlate with ancestry differences. A more effective approach to control for population stratification involves within-family analyses, where genetic differences between siblings are compared,^63,89^ though these tend to, currently, have much smaller sample sizes than population-based GWASs, and capture only direct genetic effects, excluding potentially causal indirect genetic effects (Box 3).
- ***Ancestry-related ascertainment bias*:** GWAS datasets lack global diversity,^134^ with 72% of GWAS discoveries (2005 – 2018) originating from the US, UK and Iceland.^135^ To minimize false positives from population stratification, GWASs often exclude ethnic minorities and control for small ancestry differences. This narrows the scope of GWASs on SES-related outcomes. Besides missing how SES interacts with genetics in non-Western societies, they fail to capture influential social factors within Western societies such as systemic racism or discrimination experienced by minority groups.
- ***SES-related ascertainment bias*:** People who are willing and able to participate in genetics research tend to be more healthy and have higher SES, introducing a sampling bias.^136^ UK Biobank, for example, shows smaller regional SES differences compared to census data.^3^ This bias can distort our estimates of genetic and environmental contributions to complex traits across the full SES spectrum. Additionally, collider bias may arise when participation is influenced by both genetic factors and SES, potentially distorting associations between them. Using population-based weights to adjust for the overrepresentation of healthier, wealthier participants can help correct some of the biases.^137^

## Box 3 Factors Affecting Estimation of Genetic Effects on Socio-Economic Outcomes (Part II)

### Inflation of genetic effect estimates due to socio-economic structures

A substantial part of socio-economic status (SES) is inherited through the family, household, and community one is born into, intertwining environmental and genetic influences. This can inflate genetic effect estimates in population-based GWASs. Polygenic scores for educational attainment based on population-based GWASs predict outcomes twice as strongly in children raised by biological families compared to adoptees,^138^ and ∼1.6 times more between families than within families^139^. Conducting GWASs within families^63,89^ or geographic regions^91^ significantly reduces the magnitude of genetic effects on SES-related outcomes. The three main sources of inflation of genetic effects on SES as estimated in population-based GWASs are:

- ***Indirect genetic effects***: These occur when genetic influences arise not from an individual’s own genes, but from the shared loci of family members. These effects can arise from parents (e.g., through parental investment or the prenatal environment), extended family members, or even multi-generational/dynastic effects (e.g., great-grandparents or beyond)^140^. Parents pass down half of their genes to their offspring. Polygenic scores for educational attainment constructed from untransmitted parental alleles predict socio-economic outcomes in children,^90^ likely reflecting indirect effects and/or assortative mating. Since genetic effects cluster geographically,^3^ indirect effects from individuals outside the direct family that are geographically and socially nearby could influence GWAS signals as well.^91^
- ***Gene-environment correlations***: Genes cluster within families, communities, and neighborhoods that provide favorable or unfavorable environmental circumstances. Living circumstances differ greatly between poorer and richer households and neighborhoods. Genes that have an effect on traits that influence socio-economic outcomes therefore correlate with effects that come from the environments that people are born in (passive gene-environment correlations) or are able to move to throughout their lives (active gene-environment correlations). This can make genetic effects seem stronger than they are and can induce a correlation between genetic variants and other traits (e.g., BMI and height) that are influenced by the environmental factors that come with higher or lower socio-economic outcomes.
- ***Assortative mating***: Socio-economic outcomes show strong positive assortative mating, where people select mates that resemble them. These outcomes are highly polygenic, i.e., influenced by many alleles with small effects. When trait-increasing alleles, which can be found throughout the whole genome, are transmitted together to the offspring due to assortative mating, they become increasingly correlated with each other in the population, leading to overestimation of their individual genetic effects. This is analogous to, for example, the effects of a good teacher on a student’s education being overestimated because good teachers cluster together in schools in better neighborhoods, and thus children in the class of one good teacher tend to have classes from other good teachers as well.

## References

[1] Lee JJ (2018). Gene discovery and polygenic prediction from a genome-wide association study of educational attainment in 1.1 million individuals. Nature genetics.

[2] Bulik-Sullivan B (2015). An atlas of genetic correlations across human diseases and traits. Nature genetics.

[3] Abdellaoui A (2019). Genetic correlates of social stratification in Great Britain. Nature human behaviour.

[4] Flannery K, Marcus J (2012). The creation of inequality: how our prehistoric ancestors set the stage for monarchy, slavery, and empire.

[5] Alesina A, La Ferrara E (2000). Participation in heterogeneous communities. The quarterly journal of economics.

[6] Mijs JJ (2016). The unfulfillable promise of meritocracy: Three lessons and their implications for justice in education. Social Justice Research.

[7] Wilkinson RG, Pickett KE (2009). Income inequality and social dysfunction. Annual review of sociology.

[8] Pickett KE, Wilkinson RG (2015). Income inequality and health: a causal review. Social science & medicine.

[9] Dewar M (2017). What makes a fair society? Insights and evidence.

[10] Piketty T, Saez E (2014). Inequality in the long run. Science (New York, NY).

[11] Harden KP, Koellinger PD (2020). Using genetics for social science. Nature Human Behaviour.

[12] Markovits D (2019). The Meritocracy Trap: Or, The Tyranny of Just Deserts.

[13] Dikötter F (1998). Race culture: Recent perspectives on the history of eugenics. The American Historical Review.

[14] Kevles DJ (1995).

[15] Rutherford A (2022). Control: The Dark History and Troubling Present of Eugenics.

[16] Haviland WA, Prins HE, McBride B, Walrath D (2013). Cultural anthropology: The human challenge.

[17] Llobera JR (2003). An invitation to anthropology: the structure, evolution and cultural identity of human societies.

[18] Boehm C, Boehm C (2009). Hierarchy in the forest: The evolution of egalitarian behavior.

[19] Widlok T, Tadesse WG (2005). Property and equality.

[20] Bocquet-Appel J-P (2011). When the world’s population took off: the springboard of the Neolithic Demographic Transition. Science (New York, NY).

[21] Henrich J, Boyd R (2008). Division of labor, economic specialization, and the evolution of social stratification. Current Anthropology.

[22] Weber M (1978). Economy and society: An outline of interpretive sociology.

[23] Bellah RN (2011). Religion in human evolution: From the Paleolithic to the Axial Age.

[24] Liu Y (2016). Higher education, meritocracy and inequality in China.

[25] Weatherford J (2005). Genghis Khan and the making of the modern world.

[26] Schwarz B (2005). The expansion of England: race, ethnicity and cultural history.

[27] Young MD (1994). The rise of the meritocracy.

[28] Black SE, Devereux PJ, Salvanes KG (2005). Why the apple doesn’t fall far: Understanding intergenerational transmission of human capital. American economic review.

[29] Erikson R, Goldthorpe JH, Portocarero L (1979). Intergenerational class mobility in three Western European societies: England, France and Sweden. The British journal of sociology.

[30] Hauser RM, Grusky DB (1988). Cross-national variation in occupational distributions, relative mobility chances, and intergenerational shifts in occupational distributions. American Sociological Review.

[31] Solon G (2002). Cross-country differences in intergenerational earnings mobility. Journal of Economic Perspectives.

[32] Meyer MN (2023). Wrestling with social and behavioral genomics: risks, potential benefits, and ethical responsibility. Hastings Center Report.

[33] López-Beltrán C (2007).

[34] Galton F (1891). Hereditary genius.

[35] Galton F (1883). Inquiries into human faculty and its development.

[36] Grekul J, Krahn A, Odynak D (2004). Sterilizing the “Feeble-minded”: Eugenics in Alberta, Canada, 1929–1972. Journal of Historical Sociology.

[37] Hyatt S (1997). A shared history of shame: Sweden’s four-decade policy of forced sterilization and the eugenics movement in the United States. Ind Int’l & Comp L Rev.

[38] Rutecki GW (2011). Forced sterilization of native Americans: Later twentieth century physician cooperation with national eugenic policies?. Ethics & Medicine.

[39] Liu A (2024). Evidence from Finland and Sweden on the relationship between early-life diseases and lifetime childlessness in men and women. Nature Human Behaviour.

[40] Polderman TJ (2015). Meta-analysis of the heritability of human traits based on fifty years of twin studies. Nature genetics.

[41] Furnham A, Cheng H (2013). Factors influencing adult earnings: Findings from a nationally representative sample. The Journal of Socio-Economics.

[42] Kappe R, Van Der Flier H (2012). Predicting academic success in higher education: what’s more important than being smart?. European Journal of Psychology of Education.

[43] Roth B (2015). Intelligence and school grades: A meta-analysis. Intelligence.

[44] Schmidt FL, Hunter JE (1998). The validity and utility of selection methods in personnel psychology: Practical and theoretical implications of 85 years of research findings. Psychological bulletin.

[45] von Stumm S (2020). Predicting educational achievement from genomic measures and socioeconomic status. Developmental science.

[46] Merriman C (1924). The intellectual resemblance of twins. Psychological Monographs.

[47] Rende RD, Plomin R, Vandenberg SG (1990). Who discovered the twin method?. Behavior genetics.

[48] Bouchard TJ, McGue M (2003). Genetic and environmental influences on human psychological differences. Journal of Neurobiology.

[49] Plomin R, DeFries JC, Knopik VS, Neiderhiser JM (2016). Top 10 replicated findings from behavioral genetics. Perspectives on psychological science.

[50] Engzell P, Tropf FC (2019). Heritability of education rises with intergenerational mobility. Proceedings of the National Academy of Sciences.

[51] Silventoinen K (2020). Genetic and environmental variation in educational attainment: an individual-based analysis of 28 twin cohorts. Scientific reports.

[52] Burton PR (2007). Genome-wide association study of 14,000 cases of seven common diseases and 3,000 shared controls. Nature.

[53] DeWan A (2006). HTRA1 promoter polymorphism in wet age-related macular degeneration. Science (New York, NY).

[54] Klein RJ (2005). Complement factor H polymorphism in age-related macular degeneration. Science (New York, NY).

[55] Risch N, Merikangas K (1996). The future of genetic studies of complex human diseases. Science (New York, NY).

[56] Abdellaoui A, Yengo L, Verweij KJH, Visscher PM (2023). 15 years of GWAS discovery: Realizing the promise. The American Journal of Human Genetics.

[57] Rietveld CA (2013). GWAS of 126,559 individuals identifies genetic variants associated with educational attainment. Science (New York, NY).

[58] Okbay A (2016). Genome-wide association study identifies 74 loci associated with educational attainment. Nature.

[59] Okbay A (2022). Polygenic prediction of educational attainment within and between families from genome-wide association analyses in 3 million individuals. Nature genetics.

[60] Akimova ET, Wolfram T, Ding X, Tropf FC, Mills MC (2024). Polygenic predictions of occupational status GWAS elucidate genetic and environmental interplay for intergenerational status transmission, careers, and health. Nature Human Behaviour.

[61] Hill WD (2019). Genome-wide analysis identifies molecular systems and 149 genetic loci associated with income. Nature communications.

[62] Kweon H (2025). Associations between common genetic variants and income provide insights about the socioeconomic health gradient. Nature Human Behaviour.

[63] Tan T (2024). Family-GWAS reveals effects of environment and mating on genetic associations. medRxiv.

[64] Bulik-Sullivan BK (2015). LD Score regression distinguishes confounding from polygenicity in genome-wide association studies. Nature genetics.

[65] Chen T-T (2024). Shared genetic architectures of educational attainment in East Asian and European populations. Nature Human Behaviour.

[66] Fisher RA (1918). The correlation between relatives on the supposition of Mendelian inheritance. Philosophical Transactions of the Royal Society of Edinburgh.

[67] Plomin R, Haworth CM, Davis OS (2009). Common disorders are quantitative traits. Nature reviews genetics.

[68] Visscher PM, Goddard ME (2019). From RA Fisher’s 1918 paper to GWAS a century later. Genetics.

[69] Wilding K, Wright M, von Stumm S (2024). Using DNA to Predict Education: a Meta-analytic Review. Educational psychology review.

[70] Abdellaoui A, Verweij KJ (2021). Dissecting polygenic signals from genome-wide association studies on human behaviour. Nature Human Behaviour.

[71] Darwin CR (1859). On the origin of species by means of natural selection, or the preservation of favoured races in the struggle for life.

[72] Hugh-Jones D, Abdellaoui A (2022). Human capital mediates natural selection in contemporary humans. Behavior genetics.

[73] Mills MC, Mathieson I (2022). The challenge of detecting recent natural selection in human populations. Proceedings of the National Academy of Sciences.

[74] Zeng J (2018). Signatures of negative selection in the genetic architecture of human complex traits. Nature genetics.

[75] Zeng J (2021). Widespread signatures of natural selection across human complex traits and functional genomic categories. Nature communications.

[76] O’Connor LJ (2019). Extreme polygenicity of complex traits is explained by negative selection. The American Journal of Human Genetics.

[77] Cassa CA (2017). Estimating the selective effects of heterozygous protein-truncating variants from human exome data. Nature genetics.

[78] Lek M (2016). Analysis of protein-coding genetic variation in 60,706 humans. Nature.

[79] Mathieson I (2023). Genome-wide analysis identifies genetic effects on reproductive success and ongoing natural selection at the FADS locus. Nature Human Behaviour.

[80] Ganna A (2016). Ultra-rare disruptive and damaging mutations influence educational attainment in the general population. Nature neuroscience.

[81] Ganna A (2018). Quantifying the impact of rare and ultra-rare coding variation across the phenotypic spectrum. The American Journal of Human Genetics.

[82] Gardner EJ (2022). Reduced reproductive success is associated with selective constraint on human genes. Nature.

[83] Paynter R (1989). The archaeology of equality and inequality. Annual review of anthropology.

[84] Smith ME (2010). The archaeological study of neighborhoods and districts in ancient cities. Journal of Anthropological Archaeology.

[85] Abdellaoui A (2013). Population structure, migration, and diversifying selection in the Netherlands. European journal of human genetics : EJHG.

[86] Kerminen S (2017). Fine-scale genetic structure in Finland. G3: Genes, Genomes, Genetics.

[87] Leslie S (2015). The fine-scale genetic structure of the British population. Nature.

[88] Kuznetsov IA (2023). Assessing the impact of 20th century internal migrations on the genetic structure of Estonia. bioRxiv.

[89] Howe LJ (2022). Within-sibship genome-wide association analyses decrease bias in estimates of direct genetic effects. Nature genetics.

[90] Kong A (2018). The nature of nurture: Effects of parental genotypes. Science (New York, NY).

[91] Abdellaoui A, Dolan CV, Verweij KJH, Nivard MG (2022). Gene–environment correlations across geographic regions affect genome-wide association studies. Nature genetics.

[92] Ding X, Brazel DM, Mills MC (2021). Factors affecting adherence to non-pharmaceutical interventions for COVID-19 infections in the first year of the pandemic in the UK. BMJ open.

[93] Wade L (2020). From Black Death to fatal flu, past pandemics show why people on the margins suffer most. Science (New York, NY).

[94] Dobson AP, Carper ER (1996). Infectious diseases and human population history. Bioscience.

[95] Lindahl JF, Grace D (2015). The consequences of human actions on risks for infectious diseases: a review. Infection ecology & epidemiology.

[96] Abdellaoui A (2020). Regional differences in reported Covid-19 cases show genetic correlations with higher socio-economic status and better health, potentially confounding studies on the genetics of disease susceptibility. MedRxiv.

[97] Lee RD (1987). Population dynamics of humans and other animals. Demography.

[98] Clark G, Cummins N (2015). Malthus to modernity: wealth, status, and fertility in England, 1500–1879. Journal of Population Economics.

[99] Clark G, Hamilton G (2006). Survival of the richest: the Malthusian mechanism in pre-industrial England. The Journal of Economic History.

[100] Jaadla H, Potter E, Keibek S, Davenport R (2020). Infant and child mortality by socio-economic status in early nineteenth-century England. The Economic History Review.

[101] Buss DM, Schmitt DP (2019). Mate preferences and their behavioral manifestations. Annual review of psychology.

[102] Skirbekk V (2008). Fertility trends by social status. Demographic research.

[103] Cleland J (2001). Potatoes and pills: An overview of innovation-diffusion contributions to explanations of fertility decline. Diffusion processes and fertility transition: Selected perspectives.

[104] Matysiak A, Vignoli D (2008). Fertility and Women’s Employment: A Meta-analysis: Fécondité et travail des femmes: une méta-analyse. European Journal of Population/Revue européenne de Démographie.

[105] Stulp G, Sear R, Schaffnit SB, Mills MC, Barrett L (2016). The reproductive ecology of industrial societies, Part II: the association between wealth and fertility. Human Nature.

[106] Barban N (2016). Genome-wide analysis identifies 12 loci influencing human reproductive behavior. Nature genetics.

[107] Beauchamp JP (2016). Genetic evidence for natural selection in humans in the contemporary United States. Proceedings of the National Academy of Sciences.

[108] Kong A (2017). Selection against variants in the genome associated with educational attainment. Proceedings of the National Academy of Sciences.

[109] Sanjak JS, Sidorenko J, Robinson MR, Thornton KR, Visscher PM (2018). Evidence of directional and stabilizing selection in contemporary humans. Proceedings of the National Academy of Sciences.

[110] Doepke M, Hannusch A, Kindermann F, Tertilt M (2023). The economics of fertility: A new era. National Bureau of Economics Research.

[111] Kolk M (2023). The relationship between life-course accumulated income and childbearing of Swedish men and women born 1940–70. Population Studies.

[112] Kolk M, Barclay K (2019). Cognitive ability and fertility among Swedish men born 1951–1967: evidence from military conscription registers. Proceedings of the Royal Society B.

[113] Wright S (1921). Systems of mating. III. Assortative mating based on somatic resemblance. Genetics.

[114] Horwitz TB, Balbona JV, Paulich KN, Keller MC (2023). Evidence of correlations between human partners based on systematic reviews and meta-analyses of 22 traits and UK Biobank analysis of 133 traits. Nature Human Behaviour.

[115] Schwartz CR (2013). Trends and variation in assortative mating: Causes and consequences. Annual Review of Sociology.

[116] Abdellaoui A, Verweij KJ, Zietsch BP (2014). No evidence for genetic assortative mating beyond that due to population stratification. Proceedings of the National Academy of Sciences of the United States of America.

[117] Hugh-Jones D, Verweij KJH, Pourcain BS, Abdellaoui A (2016). Assortative mating on educational attainment leads to genetic spousal resemblance for polygenic scores. Intelligence.

[118] Kemper KE (2021). Phenotypic covariance across the entire spectrum of relatedness for 86 billion pairs of individuals. Nature communications.

[119] Robinson MR (2017). Genetic evidence of assortative mating in humans. Nature Human Behaviour.

[120] Sunde HF (2024). Genetic similarity between relatives provides evidence on the presence and history of assortative mating. Nature communications.

[121] Zheng Q (2025). Genetic basis of partner choice. bioRxiv.

[122] Sunde HF, Eilertsen EM, Torvik FA (2024). Understanding indirect assortative mating and its intergenerational consequences. bioRxiv.

[123] Young AS (2023). Estimation of indirect genetic effects and heritability under assortative mating. bioRxiv.

[124] Greenwood J, Guner N, Kocharkov G, Santos C (2014).

[125] Dribe M, Lundh C (2009). Status homogamy in the preindustrial marriage market: partner selection according to age, social origin, and place of birth in nineteenth-century rural Sweden. Journal of Family History.

[126] Zijdeman RL, Maas I (2010). Assortative mating by occupational status during early industrialization. Research in Social Stratification and Mobility.

[127] Clark G (2023). The inheritance of social status: England, 1600 to 2022. Proceedings of the National Academy of Sciences.

[128] Abdellaoui A (2023). Trading social status for genetics in marriage markets: evidence from Great Britain and Norway. School of Economics Working Paper.

[129] Abdellaoui A (2022). The evolutionary dance between culture, genes, and everything in between. The Behavioral and brain sciences.

[130] Uchiyama R, Spicer R, Muthukrishna M (2022). Cultural evolution of genetic heritability. Behavioral and Brain Sciences.

[131] Tropf FC (2017). Hidden heritability due to heterogeneity across seven populations. Nature Human Behaviour.

[132] Yengo L (2018). Imprint of assortative mating on the human genome. Nature Human Behaviour.

[133] Young AI (2022). Mendelian imputation of parental genotypes improves estimates of direct genetic effects. Nature genetics.

[134] Mills MC, Rahal C (2020). The GWAS Diversity Monitor tracks diversity by disease in real time. Nature genetics.

[135] Mills MC, Rahal C (2019). A scientometric review of genome-wide association studies. Communications biology.

[136] Schoeler T (2023). Participation bias in the UK Biobank distorts genetic associations and downstream analyses. Nature Human Behaviour.

[137] van Alten S, Domingue BW, Faul J, Galama T, Marees AT (2024). Reweighting UK Biobank corrects for pervasive selection bias due to volunteering. International journal of epidemiology.

[138] Cheesman R (2020). Comparison of adopted and nonadopted individuals reveals gene–environment interplay for education in the UK Biobank. Psychological science.

[139] Selzam S (2019). Comparing Within- and Between-Family Polygenic Score Prediction. American journal of human genetics.

[140] Nivard MG (2024). More than nature and nurture, indirect genetic effects on children’s academic achievement are consequences of dynastic social processes. Nature Human Behaviour.

